# The experiences of people with type 2 diabetes in communicating with general practitioners in China – a primary care focus group study

**DOI:** 10.1186/s12875-022-01632-y

**Published:** 2022-02-03

**Authors:** Mi Yao, Dong-ying Zhang, Jie-ting Fan, Kai Lin, Shamil Haroon, Dawn Jackson, Hai Li, Wei Chen, Kar Keung Cheng, Richard Lehman

**Affiliations:** 1 Institute of Applied Health Research, University of Birmingham, Birmingham, B15 2TT UK; 2 National Clinical Research Center for Respiratory Disease, The First Affiliated Hospital of Guangzhou Medical University, Guangzhou, China; 3 Faculty of Medicine, Macau University of Science and Technology, Macau, China; 4 Department of Endocrinology, PLA Rocket Force Characteristic Medical Center, Beijing, China; 5 Family Medicine Center, The First Affiliated Hospital of Shantou University Medical College, Shantou, China; 6 College of Medical and Dental Sciences, University of Birmingham, Birmingham, UK; 7 Department of Endocrinology, The First Affiliated Hospital of Sun Yat-sen University, Guangzhou, China; 8 Department of Nephrology, The First Affiliated Hospital of Sun Yat-sen University, Guangzhou, 510 080 China

**Keywords:** Communication, General practice, Patients, Type 2 diabetes

## Abstract

**Background:**

With the implementation of health care reforms in China, primary care is on a journey to provide care for most patients with type 2 diabetes. While Chinese general practitioners (GPs) have described challenges in communication with diabetes patients in their daily practice, little is known about patients’ experiences in communicating with their GPs.

**Methods:**

Five focus groups (of 4–5 participants each) were used to explore views from patients with type 2 diabetes. Purposive sampling was used to recruit a spread of participants from general practices in Guangzhou city, China. Focus groups were audio-recorded, transcribed, and thematically analyzed using the Framework Method.

**Results:**

Ten males and 12 female patients from five general practices participated in focus group discussions, with a mean age of 57.3 years and 7.3 years of diabetes duration. Five main themes emerged: patients’ understanding about diabetes, diabetes medication, communication with GPs, physician-patient relationships, and healthcare systems and context. Patients generally searched for information on the internet, but they weren’t always sure if it was trustworthy. Several communication needs were described by diabetes patients, such as explanation of blood glucose monitoring, medication information support, communication in the risk of diabetes complications and cardiovascular disease, and language barriers. Communication was frequently brief and not tailored to their concerns, and some described being scolded or panicked by GPs. Participants acknowledged the pressures within the health system, such as short consultation times, an incoherent GP-hospital interface and high demand.

**Conclusions:**

Key issues from the patients’ perspective for the development of primary care based management of diabetes in China were identified. People with type 2 diabetes require more access to trustworthy diabetes information and wish for better channels of communication with their GPs. Strategies may be required to improve GPs’ communication skills with their patients that also consider the context of the wider health system environment in China.

**Supplementary Information:**

The online version contains supplementary material available at 10.1186/s12875-022-01632-y.

## Background

Diabetes has become a major public health problem and economic burden and is the sixth leading cause of death in China [1, 2]. It is now estimated that diabetes affects more than 140 million Chinese people, and the number is dramatically increasing [3, 4]. Diabetes patients are at increased risk of long-term microvascular and macrovascular complications including heart disease, stroke, blindness, kidney failure and extremity amputations [5, 6]. However, diagnosis, treatment, and control of diabetes are not optimal in China [7, 8]. Effective diabetes management is an urgent need in China to reduce the burden of diabetes and improve the quality of diabetes care.

The current weakness of the primary care system in China is a major barrier to optimal diabetes care [9, 10]. Primary health care in China usually does not provide the point of first contact care, and typically coordinates care under the direction of specialty care. However, care delivery systems are often fragmented between primary and secondary care [10]. There is an increasing awareness that the current diabetes care model is unsustainable. To address such issues, the Chinese government has committed to a dramatic increase in the capacity of the primary health care system, including training 400,000 new GPs in the next 10 years, and the introduction of a national essential drug system alongside a social health insurance program, introduced to improve access and affordability in primary health care [11–14]. Patients with chronic non-communicable diseases, including diabetes, will gradually transition from hospitals into primary care settings and receive care by GPs.

This qualitative focus group study is part of a programme of research, aimed to understand the communication experiences in type 2 diabetes care in China, and related training needs. A review of the literature highlighted the importance of effective communication between healthcare providers and diabetes patients to ensuring optimal diabetes care [15–17]. Also, the patient–provider relationship has a strong bearing on patients’ adherence to treatment [18, 19]. These cornerstones of care can enhance cooperation, understanding of treatment, adherence to recommendations and patient satisfaction, as well as resulting in improved clinical outcomes. Our previous focus group study suggested that Chinese GPs face challenges in communication with diabetes patients in their daily practice. They believed patients’ knowledge was insufficient and that misunderstanding was common [20]. At the same time, GPs rarely received communication skills training, which may impede effective communication with diabetes patients [20, 21].

Individualized approaches to diabetes care, taking patients’ views into account, are necessary for optimal outcomes [22, 23]. The patient perspective is critical to understanding the experience of receiving care in the current state of primary care in China. We therefore conducted our focus group study with diabetes patients to explore their experiences of communicating with their GPs and to identify elements of communication which might be improved by a GP training program.

## Method

### Study design

A qualitative study was undertaken with facilitated focus groups. The methods used were based on our previous linked study on healthcare professionals [20]. One researcher (MY) conducted all the focus groups as facilitator, and another researcher (DZ) was co-facilitator. The first author (MY, male) is a practicing general practitioner in China and undertaking a PhD in medicine in the UK, and DZ (female) is an academic researcher with relevant expertise in primary health care in China. Both researchers were trained in qualitative research and had no prior relationship with any of the participants. Each focus group was audio recorded and transcribed verbatim.

A semi-structured topic guide was used to stimulate an open conversation and to ensure key issues were covered in investigating the experience of diabetes patients in communicating with GPs during consultations. Design of the topic guide was informed by the study aim, a systematic review of literature on the impact of training healthcare professional’s communication skills on diabetes care, discussion amongst the multidisciplinary team involved in this research, and the tested before use [20, 24]. (see Table 1).

**Table 1:** Focus group discussion guide for GPs

1. Prompts for facilitators. • How did you feel when you were first diagnosed with type 2 diabetes? • What is important for you to be able to live as good a life as possible with diabetes? • Do you get the opportunity to ask questions relevant to diabetes with your GPs? • What is your experience and feeling when communicating with doctors both in hospitals and general practices? • Are there any difficulties in communicating with your GPs? • Do you like your GPs? Explain. • Do you trust your GPs? Explain. • Among the different doctors you visit, which one did you think gave you a better experience? • What do you think doctors can do to enhance communication or relationship with you during consultations? • Is there anything else about the physician/patient relationship that you want to share?	

### Recruitment

The recruitment process and focus groups took place from April to November 2020. Type 2 diabetes patients from five community health service centers (general practices) were recruited from different geographical settings (two rural and three urban districts) in Guangzhou, China. Using GP practice data, we aimed to purposively sample patients aged over 18 years, diagnosed with type 2 diabetes for at least 1 year. These inclusion criteria were chosen to enable recruitment of adult participants, who had some experience of communication interactions with GP’s about managing their diabetes. Eligible patients from 5 GP practices were recruited by an electronic letter, disseminated through each practice’s online information platform (WeChat), which introduced the research and criteria. Thirty-eight individuals expressed interest through the WeChat platform and were followed up by telephone from the research team. Of these, 22 consented and participated in focus groups. All focus groups were held in the general practices where participants were registered for their convenience. All provided written informed consent and completed a questionnaire to collect demographic information including age, gender, years of diabetes, education background, status of employment, hypertension, and diabetes pharmacotherapy. None of the participants were known to the interviewers. No repeat interviews were carried out. We stopped patient recruitment when data saturation was reached. Subsequent analysis did not identify significant new codes, views, or experiences, so it was concluded that data saturation had been achieved. A compensation of a RMB 200 (equivalent to 30 US dollars) shopping voucher was offered to participants for the costs of travel. Approval was obtained from the Medical Ethics Committee of The First Affiliated Hospital of Sun Yat-sen University (Reference number [2019]369).

### Analysis

Audio-recorded data were professionally transcribed and reviewed for accuracy by two researchers (MY & JF). One focus group transcript was randomly selected by researchers and returned to participants for comments within 2 days of the group discussion to check the accuracy of the transcription. Following participant checking, no corrections were required for this transcript. Anonymized transcripts and field notes were imported into NVivo12 software and coded independently by two researchers (MY & DZ).

The Framework Method was used for thematic analysis, aligning to our analytic approach elsewhere in our programme of research [20, 25]. Analysis was ongoing and iterative, informing further data collection. This included the incorporation of new insights from focus group participants to refine the interview topic guide, and to inform the Framework Method of our analysis. For the first stage of the thematic analysis, two researchers (MY and DZ) independently read two random focus group discussion transcripts (and associated field notes) and open-coded the data by marking and categorizing key words and phrases to generate initial codes. These were discussed, and discrepancies resolved through consensus to develop the initial thematic framework, which was then applied to the remaining transcripts.

Once all the data had been coded using this framework, we summarized the data in a matrix based on similarities and differences of codes. Sub-themes were generated from the data set by reviewing the matrix and making connections within codes. Themes and sub-themes were identified until data saturation was confirmed in the analysis. The analysis and interpretations of the data were discussed by researchers (MY and DZ) until they reached a consensus. The findings were provided to four participants (from two focus groups) for review, and the participants were in agreement with the interpretation of the research team.

The Consolidated Criteria for Reporting Qualitative Research (COREQ) checklist was used when writing this report [26]. (see additional file).

## Results

In total, we ran five focus group discussions with 22 diabetes patients, with a mean age of 57.3 years, having lived with diabetes for an average of 7.3 years. The mean duration of focus groups was 65 min (range 55 to 80 min), no participant dropped out. Details of participant and focus group characteristics are provided in Table 2.

**Table 2:** Focus group characteristics, *N* = 22

Characteristic	n
**Age**	
Mean (SD), years	57.3 (10.8)
**Sex ratio**	
Male: female	10:12
**Duration of diabetes**	
Mean (SD), years	7.3 (5.1)
**Education background**	
Junior high school or below	12
High school	7
College or above	3
**Status of employment**	
Working	10
Retired	12
**Hypertension**	
Yes	8
No	14
**Diabetes pharmacotherapy**	
Diet alone only	1
Oral medication only	16
Oral medication and insulin injections	5
**Location**	
City center	14
Rural or suburb	8

Five main themes were conceptualised from the group discussions: patients’ understanding about diabetes, diabetes medication, communication with GPs, physician-patient relationships, and healthcare systems and context. The themes and subthemes are presented in Table 3.

**Table 3:** Themes and subthemes

Themes	Subthemes
1. Patients’ understanding of diabetes	a. Impact of diabetes
	b. Sources of knowledge
2. Diabetes medication	a. Medication information support
	b. Adherence to oral hypoglycemic agents
	c. Traditional Chinese medicine & herbal medicines
3. Communication with GPs	a. Blood glucose measurement and monitoring
	b. Risk of diabetic complications and cardiovascular disease (CVD)
	c. Poor and good communication experiences
	d. Language barriers
4. Physician-patient relationships	a. What constitutes a good physician
	b. Sympathy to GPs’ busy clinical work
	c. Building relationships with physicians
	d. Personal responsibility
5. Healthcare systems and context	a. Diagnosis and hospitalization
	b. Convenience of community health service centers
	c. Environment of the consultation room

### Theme 1: patients’ understanding of diabetes

1a. Impact of diabetes.

Patients described a significant impact of diabetes from the time of initial diagnosis to living with the condition in the long term. Some described that they were unexpectedly diagnosed with diabetes through screening and health check-ups as they did not have any symptoms. They expressed initially experiencing denial of the diagnosis, fear, depression, anxiety and worry. As time went on, various symptoms and complications troubled most of them, such as fatigue, weight loss, hypoglycemia, and itchy skin. A few patients described challenges in living with a diabetes label, especially in their work and social activities. Patients also worried about their diet and life-style changes, organ impairment and comorbidities. They had doubts and questions about the best treatments for diabetes, and whether diabetes was inherited in their families.

‘When I was first diagnosed with diabetes, I felt as if I was sentenced to death. How can a person suddenly become like this? I can't accept it.’ (FG [focus group]2 P1)

‘Words jump out of my head that I am a chronic disease patient who cannot eat more. I always think of myself as a diabetes person.’ (FG4 P2)

#### 1b. Sources of knowledge

Patients described several ways of acquiring knowledge about diabetes and other health-related issues. They believed that information from friends, family members and other diabetes patients was useful, and trustworthy. They also searched for information from the Internet and social media, such as WeChat (a popular mobile phone social application in China) and Tik Tok (a popular mobile phone short video application). However, they found it difficult to judge whether the information presented on these platforms was trustworthy, whilst some information made them more worried about their condition. They reported rarely receiving health information from GPs in clinical encounters. However, they mentioned that they did get information through health education classes in community health care settings. This typically consists of a group lecture or class for 50–100 patients, usually administered by a doctor or nurse.

‘It's usually my relatives and friends with diabetes who talk too much about diabetes information. Doctors rarely tell me this.’ (FG1 P4)

‘Sometimes I look for information online, but it is just made up. I am afraid that the information is false. I neither believe nor know how to judge.’ (FG3 P3)

### Theme 2: diabetes medication

2a. Medication information support.

Patients felt that they needed medication information support and more communication with their GPs. Several patients expressed particular concerns about the comparison of effectiveness between drugs, differences between generic and branded drugs, adverse drug reactions (such as hypoglycemia), information about new drugs (such as sodium-glucose cotransporter-2 (SGLT2) inhibitors) as well as the price of different drugs. Patients wanted their GPs to advise them on medication therapies with more detail, such as the indications for medicines and rationale for changing or stopping medications. However, they frequently felt that almost no GPs, nurses or pharmacists in clinics gave such information. Patients also hoped that GPs would explain more about the complexities of diabetes therapies in combination with other treatments, such as statins or antiplatelet drugs. Again, they felt this information from GPs was lacking.

‘I asked my doctor if there were many side effects. What is bad for the stomach and intestines? My stomach is very upset, so whether I can take the medicine less once a day?’ (FG4 P1)

‘The doctor said that this medicine was rather expensive. A box of medicine costs more than 60 RMB. Health insurance does not cover it. The price of my other drugs adds up to nearly 200 RMB, which can be reimbursed. If this cannot be reimbursed, I may not accept it.’ (FG2 P3)

‘The doctor didn't give me a good explanation of what they were and why I was taking them.’ (FG5 P4)

2b. Adherence to oral hypoglycemic agents.

Patients described fears, concerns, and distress regarding oral hypoglycemic agents. They did not wish to take medication when first diagnosed with diabetes. They believed that once started, they would need to take these lifelong. Some patients said taking medication before or after meals made them feel embarrassed when eating with friends or family members, and described instances when they would find an excuse to leave and take their medication. Some patients recognized the importance of medication concordance. However, others (frequently older participants, or those with diabetes for a longer duration) described difficulties in taking multiple medications, and found they often got these mixed up or forgot to take them.

‘After all, I'm not yet 40. I am still young. I'll try to put off taking the medicine.’ (FG3 P1)

‘I probably don't take my medicines on time, and sometimes I forget to take.’ (FG1 P2)

               ‘I’m embarrassed to tell people why I take medicines when having lunch with them.’ (FG4 P3)

‘I take 7 or 8 different medications and sometimes I can't tell them apart. I feel like I become stupid if I take too many.’ (FG2 P4)

2c. Traditional Chinese medicine & herbal medicines.

A few patients described trying traditional Chinese medicine (TCM) and herbal medicines when first diagnosed with diabetes. Most information on TCM therapies came from other diabetes patients, rather than GPs. One patient believed his diabetes could be reversed by TCM. Some patients would take TCM while concurrently taking western medications. Compared with western medication, patients saw TCM as supplements without any side effects. A few patients believed that TCM could make narrow blood vessels more open. However, others felt that they took TCM but saw no effect in the control of their condition.

‘When I was first diagnosed, I heard from friends with diabetes that there would be a lot of sequelae and trouble after taking the western medicine. So, I tried traditional Chinese medicine and I felt my body function start to recover a little bit.’ (FG3 P1).

‘After all, traditional Chinese medicine has no side effects and does less harm to the body. Besides, some traditional Chinese medicine can open blood vessels.’ (FG5 P1).

‘I went to see a traditional Chinese doctor. But after taking traditional Chinese medicine, I didn’t see any real effect.’ (FG5 P3).

### Theme 3: communication with GPs

3a. Blood glucose measurement and monitoring.

Most participants, across every focus group, described the importance of blood glucose figures and monitoring, such as fasting and postprandial blood glucose, and HbA1c. Higher figures or transient fluctuations figures made them worry about their condition and eager to discuss their results with doctors. They saw normal blood glucose figures as an indicator of stable status in diabetes management. Some patients even described experiences of their self-confidence coming back when higher figures returned to normal. A number of patients described instances when their GPs had set goals for self-monitoring of their blood glucose, though they usually did this less frequently than recommended.

‘Get those blood glucose levels down to normal, and you'll be fine. Or you're really upset.’ (FG2 P4)

'As long as the blood glucose comes down, I will be confident.' (FG2 P1)

‘My blood glucose fluctuates a lot, then I go to see my doctor for help.’ (FG5 P3)

‘The doctor told me the goal and self-monitoring at home, but I rarely did it.’ (FG1 P3)

3b. Risk of diabetes complications and cardiovascular disease.

Most patients were concerned about diabetes complications, especially eye problems, kidney problems, and amputation. They had heard about diabetes complications from their doctors, family members with diabetes or other diabetes patients. They mentioned that their doctors simply required them to control their blood glucose within normal range alongside self-observation for symptoms. However, they expressed that further information was needed from GPs on how diabetes could progress to complications. Almost no patients in focus groups mentioned cardiovascular disease (CVD) in their discussions. When CVD was suggested by the group facilitators, almost no patients recognized that diabetes could increase the risk of CVD, and reflected that they had not been informed of this information, even by GPs.

‘I have a relative who has diabetes. He has lost his eyesight, problems with his kidneys and liver. He almost has problems with his whole body. Although he had been hospitalized, but nothing worked. He was miserable. He could not sleep one night because of the pain. He was not very old, and just in his 50s.When I thought about him, and then realized that I had diabetes myself, I was particularly afraid of these complications. I wish I had a doctor to talk to me about these things.’ (FG3 P3)

‘When I first came to see my doctor, he said something about the complications of diabetes, but then he didn't say anything more in following visits. I was told to watch my blood glucose and pay more attention.’ (FG5 P2)

‘Doctors neither told me about heart disease or stroke, nor the information that diabetes can increases the risk of such disease.' (FG4 P2)

3c. Poor and good communication experiences.

Some participants believed that GPs attempted to persuade patients, for example by leading them to panic about severe complications of diabetes by using dramatic illustrations, such as pictures of amputation. Communication with GPs was frequently described as very brief, sometimes without any words, and with no explanation of recommended therapies. One patient mentioned that her doctor used clinical guidelines to persuade her to follow advice. Another participant reflected that he was scolded by his GP for asking more questions. He felt his doctor was unhappy and the interaction resulted in the provision of a prescription without explanation. Most patients hoped that their GPs would give them more guidance about diet, exercise, medication, and ways to access resources for support and diabetes education. They also wanted to have more options to access channels of patient-doctor communication, rather than just clinical encounters in their GP appointments.

Not all experiences were poor, and there were some good communication experiences described by patients, including those who felt their views were respected by their GP, through prompt provision of feedback, the use of clear and frank words and positive body language (such as touch or delivering paper towels to wipe tears). Many patients also mentioned good communication experiences in telephone calls and online communication with their GP. Patients had the ability to add their GPs to their contacts through Wechat and joined in diabetes patient online groups through this option, which typically consisted of an online chat within around 300–500 patients. Some patients felt they could easily and quickly ask questions on this online forum (Wechat), and their GPs would respond.

‘He (the GP) said this was what the treatment guideline shows and how it should be followed.’ (FG2 P3)

‘He (the GP) showed me a picture with a diabetes patient lose one leg. And he said if you did not control diabetes and you might be like that patient.’ (FG2 P4)

‘Sometimes the doctor scolds me for asking too much.’ (FG4 P4)

‘Once he took my hands and said you did not be afraid. That was really touching. I think he is a good doctor- better than my son.' (FG4 P3)

‘Call him (the GP) when you don't feel well in the evening and he's always there to answer you’ (FG1 P1)

‘I always added GPs to my WeChat contacts and asked them questions. I also read diabetes information that GPs sent out to other patients in the WeChat group.’ (FG1 P2)

3d. Language barriers.

Some patients described language barriers in their communication with GPs. Some were not fluent in speaking or understanding Mandarin (the official language in China, and typically used in professional communication) and their GPs also had difficulties in understanding patients’ local dialects. Patients wished to visit doctors who spoke the same language as them. Some found it difficult to have relatives to accompany them to provide translation support. In addition to these concerns, some patients wanted their GPs to use common or plain language, rather than medical terminology.

‘Sometimes I have questions, but I can't express them in Mandarin.’ (FG2 P4)

‘Cantonese is easy for me to understand and express. If the doctor speaking Mandarin, I can't understand what he says.' (FG2 P1)

‘Doctors should be wise. They should say something common, then everyone will understand.’ (FG5 P3)

### Theme 4: physician-patient relationships

Almost all the focus groups participants mentioned desirable traits of an ‘ideal’ or ‘good’ physician, which included a caring attitude, patience, responsibility, listening to patients, alongside active feedback, and the ability to solve patients’ problems. However, when asked how they chose their GPs, they frequently made this judgement based on the doctor’s educational background and recommendations from family and friends. They frequently liked to build a relationship with one ‘good’ GP for a long time, though a few patients liked to randomly visit doctors (both specialists and GPs) rather than to build a long-term relationship with them.

‘The one who is very careful, very kind, and caring for me, and who can make me feel comfortable is a good doctor’ (FG1 P2)

‘A good doctor can solve the patient's problems professionally, listen to the patient carefully, and make the patient feel comfortable.’ (FG5 P2)

Many of the participants expressed a sympathy and appreciation of GPs’ busyness and hard work. They were aware that doctors saw large numbers of patients every day and consultation time with them was very short. Some felt that they should not take up too much of their doctor’s time during clinical encounters and felt they should cooperate with doctors as much as possible to decrease their burden.

‘GPs see a lot of patients. I do not talk to him for long. I do not want to burden them by taking up too much of their time. They already work very hard.’ (FG4 P3)

Some participants hoped that their GPs would take over control of their condition and remind them what to do and not do. They felt the responsibility of diabetes management should sit with their GPs. However, others disagreed with this opinion and believed that it was the patient’s own responsibility.

‘I will take my GP's advice. It would be better if he kept pushing me. I wish he could fully manage my diabetes.’ (FG5 P1)

### Theme 5: health care systems and context

Many of the participants had experienced diabetes care in both hospital and GP settings. Some had been admitted to hospital to facilitate their diabetes diagnosis, especially when glucose figures were detected to be abnormal through screening or health check-ups by GPs or specialists. They were also hospitalized by specialists to control their blood glucose, dramatically change their medication or for a full ‘check’ for diabetes complications, frequently comprising a range of imaging and blood tests.

‘When I was first diagnosed with diabetes, I was admitted directly to a hospital for diagnosis and treatment.’ (FG1 P3)

‘I was hospitalized routinely once every two years for CT, B-ultrasound, neurological test, as well as examination of all organs of the body, such as heart, lung and liver. It costed more than 10,000 RMB each time, and then stayed in hospitals for five to seven days without any treatment effect.’ (FG4 P3)

Patients described that they were free to visit different specialists or GPs as they chose, and they subsequently compared the advice from these different sources. Official referral routes between hospital and primary care were rarely described, and navigation between services was frequently initiated by the patients themselves. Experiences in seeking care from hospitals was generally thought to be worse, including long travel, crowded clinics, difficulties in obtaining appointments, short consultations, financial costs, and the recommendation of complex or costly procedures and tests felt to be irrelevant to their condition.

Participants described the convenience of GP care, such as the ability to walk-in without an appointment, less crowding, less reliance on complex procedures, and being cheaper and easier for multiple prescriptions. They also felt that GPs could solve other health issues in addition to diabetes problems. Participants in both rural and suburban discussion groups hoped that the environment of GP consultation rooms could improve to include one patient with one doctor in one consultation room rather than crowded patients with more than two doctors in one room. Participants found that primary care medication lists often did not match those from the hospital, and they wanted a system where these could be aligned to avoid unnecessary changes or confusion.

‘It's very convenient to visit a general practice, and you can come at any time. It is quick to get my prescription. Also, other health problems can be solved at the same time’ (FG3 P2)

‘Of course, we want the environment of general practices to be better. Instead of having a room full of doctors and patients, either each patient or doctor has a separate room.’ (FG1 P4)

‘There is not a wide range of medicines available in general practices. Sometimes drugs that are given in hospitals are not available in general practices. I don't want to change my current medication.’ (FG3 P2)

## Discussion

In this study, we explored diabetes patients’ experience in communicating with their GPs in China. The rich information from focus group discussions has illuminated several important areas for consideration. Several of the needs described by diabetes patients in communication with GPs, such as medication information support, communication of risk, complications and CVD.

Compared with our previous focus group study with GPs, we found that the patient participants shared the same health system concerns as the GP participants, including short consultation times and difficulties in accessing trustworthy diabetes information [20]. Such challenges can impede effective communication between GPs and diabetes patients and indicate that good doctor-patient communication requires a sufficiently resourced healthcare environment to support it. Improving communication with patients in China is therefore likely to also require contextual changes to lead to meaningful change. Our results suggest this may require consideration of appointment duration, consultation environments, communication channels between specialists and GPs, the way in which funding and cost are administered and access to trustworthy information.

To our surprise, and in contrast to our previous study with GPs, the patient groups frequently expressed sympathy and appreciation of GPs’ busy clinical work rather than “blaming doctors” [20]. Instead, they appeared to attribute communication difficulties to the pressures of short consultation times and frequently made concessions for this in their communication expectations. Despite the reality of many of the described experiences, patients hoped their GP would be kind, caring and problem-solving. Although patients acknowledged that communication between each side was inadequate, experiences of remote or online communication encounters was typically felt to be good. Online platforms were considered to provide more time and space for patients to ask questions and acquire tailored information and offer novel approaches for doctor-patient communication beyond the typical in-person clinical interaction.

Stories of scolding and panicking patients were unexpected and pose a significant risk to patient-doctor communication and to diabetes care. These experiences signify the need for attitudinal change, highlighting the importance of the patient perspective, the creation of space for patient questions and their active involvement in plans about their care [15]. These also suggest a need for clinical skills training. These negative experiences may reflect the previously described pressures of the busy clinical environment, and training programmes will need to consider the socio-cultural context in their design.

Blood glucose control was a specific focus for many of the patients in our study. This frequently appeared to be the goal of diabetes care, and an obvious and easy indicator for patients. However, transient fluctuations in blood glucose caused patients uneasiness, worry and often drove additional consultation with GPs. We found that communication of diabetes complications and risks was frequently sparse, and particularly rare when considering CVD risks. These long-term goals in diabetes care represent important areas for clinical care, and should not be neglected [5]. Communication skills training for GPs in China should ensure that such areas are addressed, and patient tools and communication aids may further facilitate these conversations. Communication of this type must also offer a tailored discussion, adapting to the particular risk profile of the patient and providing relevant management advice that takes the patient perspective into account [17, 27].

Compared with previous studies in developed countries, similar themes emerged from our study. Those themes were impact of illness [28], knowledge and information needs [29], medication adherence [30], seeking alternative therapies [28], and access to healthcare settings [31]. Several of the experiences in poor communication skills and barriers in communication reported in our study were also consistent with one systematic review [24]. However, there are some differences in themes which could be explained by the context of primary care in China. For example, diabetes was frequently diagnosed and treated initially through hospitalization. We also would suggest that the access that patients have to GPs on online discussion forums may not be available in other countries. Such differences further emphasize the need for experiences in China to be studied, so that training programme design and support for communication reflect local needs and healthcare context.

To our knowledge, this is the first study to describe experiences of type 2 diabetes patients in their communication with GPs in China. One limitation of the study is that the sample was drawn from a single city in China, and it is possible that the views and experiences of patients from other geographic regions would differ. However, purposive sampling was used in our focus groups to encompass a range of patients, across both urban and rural general practices, ages, and duration of diabetes. Our focus groups were small groups. We used smaller groups due to the complexity of the topic and a desire for more in-depth insights from participants. However, these smaller groups also provided the advantage of being easier to recruit and host, providing more opportunity to share ideas, being more comfortable for participants and having less fragmentation of discussion compared with larger groups [32]. Combined with our previous focus group study with GPs, this study presents a picture of communication between diabetes patients and GPs in China, which will benefit future research and policymaking for improving this area.

## Conclusion

Key issues from the patients’ perspective for the development of primary care based management of diabetes in China were identified. These provide a starting point for planning a viable transition from secondary to primary care and also a baseline from which to assess progress. The challenges are considerable. Success in the long term management of diabetes depends on patient understanding and self-management, and the picture that emerges from our study is that these needs are currently very poorly addressed. China has a fast-developing knowledge-based economy, and the information needs of patients should be relatively easy to meet, provided that this is done in a structured way that meets all levels of literacy and is tailored to each locality’s health system and languages. Our study reveals that many patients have little confidence in their ability to get timely advice from health professionals, and sometimes receive conflicting advice. Even with a massive expansion of the primary care medical workforce, general practitioners alone cannot address all the support needs of the 150 million Chinese patients with diabetes but will need to be augmented by multi-professional teams working at grass roots level. Such basic changes in the quality of communication and the structure of care offer the prospect of greatly improved outcomes over the lifetime of this population.

## Supplementary Information

**Additional file 1.**

## Data Availability

The anonymized transcribed data from the current study are available from the corresponding author on reasonable request.

## References

[1] National Health Commission of the People's Republic of China (2019). China health statistical yearbook 2019.

[2] Wang W, McGreevey WP, Fu C, Zhan S, Luan R, Chen W, Xu B (2009). Type 2 diabetes mellitus in China: a preventable economic burden. Am J Manag Care.

[3] Wang L, Gao P, Zhang M, Huang Z, Zhang D, Deng Q, Li Y, Zhao Z, Qin X, Jin D (2017). Prevalence and ethnic pattern of diabetes and Prediabetes in China in 2013. JAMA.

[4] Li Y, Teng D, Shi X, Qin G, Qin Y, Quan H, Shi B, Sun H, Ba J, Chen B (2020). Prevalence of diabetes recorded in mainland China using 2018 diagnostic criteria from the American Diabetes Association: national cross sectional study. BMJ.

[5] Buse JB, Ginsberg HN, Bakris GL, Clark NG, Costa F, Eckel R, Fonseca V, Gerstein HC, Grundy S, Nesto RW (2007). Primary prevention of cardiovascular diseases in people with diabetes mellitus. Diabetes Care.

[6] Tuttle KR, Bakris GL, Bilous RW, Chiang JL, de Boer IH, Goldstein-Fuchs J, Hirsch IB, Kalantar-Zadeh K, Narva AS, Navaneethan SD (2014). Diabetic kidney disease: a report from an ADA consensus conference. Diabetes Care.

[7] Ji L, Hu D, Pan C, Weng J, Huo Y, Ma C, Mu Y, Hao C, Ji Q, Ran X (2013). Primacy of the 3B approach to control risk factors for cardiovascular disease in type 2 diabetes patients. Am J Med.

[8] Jia W, Weng J, Zhu D, Ji L, Lu J, Zhou Z, Zou D, Guo L, Ji Q, Chen L (2019). Standards of medical care for type 2 diabetes in China 2019. Diabetes Metab Res Rev.

[9] Li X, Lu J, Hu S, Cheng KK, De Maeseneer J, Meng Q, Mossialos E, Xu DR, Yip W, Zhang H (2017). The primary health-care system in China. Lancet (London, England).

[10] Li X, Krumholz HM, Yip W, Cheng KK, De Maeseneer J, Meng Q, Mossialos E, Li C, Lu J, Su M (2020). Quality of primary health care in China: challenges and recommendations. Lancet.

[11] General Office of the State Council of the People’s Republic of China: Guidance on setting up hierarchical medical system. 2015. Date accessed: June 30 2021. [http://www.gov.cn/zhengce/content/2015-09/11/content_10158.html].

[12] Lian S, Chen Q, Yao M, Chi C, Fetters MD (2019). Training pathways to working as a general practitioner in China. Fam Med.

[13] General Office of the State Council of the People’s Republic of China: Opinions of the general office of the state council on reforming and improving general practitioner training and incentive mechanisms. 2018. Date Accessed: 30 June 2021. [http://www.gov.cn/zhengce/content/2018-01/24/content_5260073.html].

[14] Meng Q, Fang H, Liu X, Yuan B, Xu J (2015). Consolidating the social health insurance schemes in China: towards an equitable and efficient health system. Lancet.

[15] Naik AD, Kallen MA, Walder A, Street RL (2008). Improving hypertension control in diabetes mellitus: the effects of collaborative and proactive health communication. Circulation.

[16] Schoenthaler AM, Schwartz BS, Wood C, Stewart WF (2012). Patient and physician factors associated with adherence to diabetes medications. Diabetes Educ.

[17] Yao M, Zhou X-Y, Xu Z-J, Lehman R, Haroon S, Jackson D, Cheng KK (2021). The impact of training healthcare professionals’ communication skills on the clinical care of diabetes and hypertension: a systematic review and meta-analysis. BMC Fam Pract.

[18] Zolnierek KB, Dimatteo MR (2009). Physician communication and patient adherence to treatment: a meta-analysis. Med Care.

[19] Ciechanowski PS, Katon WJ, Russo JE, Walker EA (2001). The patient-provider relationship: attachment theory and adherence to treatment in diabetes. Am J Psychiatr.

[20] Yao M, Zhang D-Y, Fan J-T, Lin K, Haroon S, Jackson D, Li H, Chen W, Lehman R, Cheng KK (2021). The experiences of Chinese general practitioners in communicating with people with type 2 diabetes—a focus group study. BMC Fam Pract.

[21] Liu X, Rohrer W, Luo A, Fang Z, He T, Xie W (2015). Doctor-patient communication skills training in mainland China: a systematic review of the literature. Patient Educ Couns.

[22] Type 2 diabetes in adults: management. 2020. Date accessed: June 30 2021. [https://www.nice.org.uk/guidance/ng28/chapter/Recommendations#individualised-care].

[23] Inzucchi SE, Bergenstal RM, Buse JB, Diamant M, Ferrannini E, Nauck M, Peters AL, Tsapas A, Wender R, Matthews DR: Management of Hyperglycemia in Type 2 Diabetes: A Patient-Centered Approach: Position Statement of the American Diabetes Association (ADA) and the European Association for the Study of Diabetes (EASD). Diabetes Care. 2012;35(6):1364-79. 10.2337/dc12-0413 PMC3357214 22517736

[24] Peimani M, Nasli-Esfahani E, Sadeghi R (2020). Patients' perceptions of patient-provider communication and diabetes care: a systematic review of quantitative and qualitative studies. Chronic Illn.

[25] Gale NK, Heath G, Cameron E, Rashid S, Redwood S (2013). Using the framework method for the analysis of qualitative data in multi-disciplinary health research. BMC Med Res Methodol.

[26] Tong A, Sainsbury P, Craig J (2007). Consolidated criteria for reporting qualitative research (COREQ): a 32-item checklist for interviews and focus groups. Int J Qual Health Care.

[27] Nano J, Carinci F, Okunade O, Whittaker S, Walbaum M, Barnard-Kelly K, Barthelmes D, Benson T, Calderon-Margalit R, Dennaoui J (2020). A standard set of person-centred outcomes for diabetes mellitus: results of an international and unified approach. Diabet Med.

[28] Lin C-C, Anderson RM, Hagerty BM, Lee B-O. Diabetes self-management experience: a focus group study of Taiwanese patients with type 2 diabetes. 2008;17(5a):34-42. 10.1111/j.1365-2702.2007.01962.x 18093120

[29] Matthews SM, Peden AR, Rowles GD. Patient–provider communication: Understanding diabetes management among adult females. Patient Educ Couns. 2009;76(1):31-7. 10.1016/j.pec.2008.11.022 19157762

[30] Brundisini F, Vanstone M, Hulan D, DeJean D, Giacomini M (2015). Type 2 diabetes patients’ and providers’ differing perspectives on medication nonadherence: a qualitative meta-synthesis. BMC Health Serv Res..

[31] Carolan M, Holman J, Ferrari M. Experiences of diabetes self-management: a focus group study among Australians with type 2 diabetes. 2015;24(7-8):1011-23. 10.1111/jocn.12724 25363710

[32] Richard A (2015). Krueger MAC: focus groups: a practical guide for applied research.

